# Revolution in Anæsthetics

**Published:** 1860-03

**Authors:** 


*A Revolution in Anæsthetics.*

The Paris journals describe a new method of producing insensibility, or rather a new way of applying an old method, which may be available in some cases, but which must frequently fail. We copy from the *Lancet* the following account of the process:

"The patient, either sitting up or lying down, is put in a convenient position. The operator then, standing either before or behind him, places before his eyes, at the distance of a few inches, but generally nearer than the point which allows of distinct vision, some bright object, upon which the patient should steadily and continuously fix his eyes. The bright object should be so placed that the eyes, in looking at it, must be forcibly directed upward, the contraction of the superior recti being carried to its maximum degree. In this position, the levatores palpebrarum and recti are strongly contracted, and convergent strabismus takes place. After this attitude, which is certainly very fatiguing, has been kept up for two or three minutes, the pupils are noticed to contract, and soon afterwards to dilate; the eyelids quiver rapidly, then fall, and the patient is asleep. Two symptoms, almost always present, are then observed; they are however, in different cases, more or less marked and lasting: 1 catalepsy, exactly as described in books; 2, anæsthesia, which lasts from three to fifteen minutes, either complete or incomplete, but which allows of pinching, pricking, and tickling, without any feeling being aroused in the patient, and without any change in the cataleptic state being produced. This anæsthetic state is generally followed by a very opposite condition—namely, very remarkable hyperæsthesia, in which the senses, the feeling of heat and muscular activity reach an unusual degree of excitability. At any moment of the experiment the symptoms may suddenly be stopped, by rubbing the eyelids, and directing upon them a stream of cold air. When the patients recover their senses, they remember nothing of what has taken place."

This is evidently nothing more than the phenomenon which is called Mesmerism, or animal magnetism, long known, little understood and frequently brought before the public as something new. The best account of it is to be found in the Five Essays of the late Dr. Mitchell, of Philadelphia.

